# ERGA-BGE reference genome of the lineid heteronemertean *Lineus lacteus *(Pilidiophora, Nemertea)

**DOI:** 10.12688/openreseurope.21501.2

**Published:** 2025-11-25

**Authors:** Aida Verdes, Patricia Alvarez-Campos, María Conejero, Ana Riesgo, Astrid Böhne, Rita Monteiro, Javier Palma-Guerrero, Rosa Fernández, Marta Gut, Laura Aguilera, Francisco Câmara Ferreira, Fernando Cruz, Jèssica Gómez-Garrido, Tyler S. Alioto, Chiara Bortoluzzi

**Affiliations:** 1 Department of Biodiversity and Evolutionary Biology, Calle José Gutiérrez Abascal 2, National Museum of Natural Sciences (MNCN-CSIC), Madrid, 28006, Spain; 2 Department of Biology, Calle Darwin 2, Autonomous University of Madrid, Madrid, 28049, Spain; 3 Leibniz Institute for the Analysis of Biodiversity Change, Adenauerallee 127, Museum Koenig Bonn, Bonn, 53113, Germany; 4 Research Institute of Organic Agriculture, Ackerstrasse 113, FiBL, Frick, 5070, Switzerland; 5 Metazoa Phylogenomics Lab, Passeig marítim de la Barceloneta 37-49, Institute for Evolutionary Biology (CSIC-UPF), Barcelona, 08003, Spain; 6 Centro Nacional de Análisis Genómico (CNAG), Baldiri Reixac 4, Barcelona, 08028, Spain; 7 Universitat de Barcelona (UB), Barcelona, 08028, Spain; 8 SIB Swiss Institute of Bioinformatics, Amphipôle, Quartier UNIL-Sorge, Lausanne, 1015, Switzerland

**Keywords:** Lineus lacteus, Lineidae, genome assembly, European Reference Genome Atlas, Biodiversity Genomics Europe, Earth Biogenome Project, nemertean, ribbon worm

## Abstract

The reference genome of *Lineus lacteus* is a crucial resource for studying the genetic basis of novelty and the evolution of remarkable traits, such as regeneration and venom, as well as the molecular mechanisms underlying adaptability in marine intertidal ecosystems. *Lineus lacteus* belongs to the Nemertea, a phylum of worm-shaped animals comprising approximately 1,300 species within the Lophotrochozoa — a superphylum of animals including leeches, snails, and other invertebrates that is crucial to our understanding of bilaterian evolution. Despite their evolutionary and ecological relevance, genomic resources for the phylum Nemertea remain scarce. We assembled the entirety of the *L. lacteus* genome into 19 contiguous chromosomal pseudomolecules. This chromosome-level assembly encompasses 0.37 Gb, composed of 71 contigs and 27 scaffolds, with contig and scaffold N50 values of 8.9 Mb and 20.4 Mb, respectively.

## Introduction

*Lineus lacteus*, a member of the Lineidae family, is a marine ribbon worm species primarily characterized by its unique morphological and genetic characteristics. The species can reach lengths of up to 60 cm but is only 1–2 mm wide. The body colour is milky white, with a reddish anterior part. The head is elongated and has 6 to 15 eyes on each side arranged in a dorsolateral row. The mouth is located ventrally, far behind the cerebral ganglia, unlike other lineids in which it is much closer ( Ament-Velásquez *et al.*, 2016; Bachiller, 2016; Rathke, 1843). The species is distributed throughout the Mediterranean Sea and the North Atlantic Ocean.

Ribbon worms are major predators in marine ecosystems, feeding on species at the top of the food chain, such as predatory annelids and crustaceans, as well as commercially important species like oysters and mussels. Nemerteans use toxins to paralyze their prey and secrete a toxic mucus to defend themselves from predators ( Göransson *et al.*, 2019). In addition, many nemerteans have remarkable regenerative capabilities, being able to regrow entire body parts and in some cases, even a whole individual from a small fragment of the body ( Zattara *et al.*, 2019).

Developing a high-quality reference genome for *Lineus lacteus* is essential for advancing our understanding of its unique genetic characteristics and adaptive traits. This genomic resource will provide valuable insights into the molecular ecology of an important predator in marine ecosystems and the evolutionary processes that shape venom composition and toxin diversity within the Nemertea, offering broader implications for venomics and toxinology research. In addition, *L. lacteus* belongs to the Lophotrochozoa — a superphylum of animals including leeches, snails and other invertebrates that is crucial to our understanding of bilaterian evolution.

The generation of this reference resource was coordinated by the European Reference Genome Atlas (ERGA) initiative’s Biodiversity Genomics Europe (BGE) project, supporting ERGA’s aims of promoting transnational cooperation to promote advances in the application of genomics technologies to protect and restore biodiversity ( Mazzoni *et al.*, 2023).

## Materials & methods

ERGA's sequencing strategy includes Oxford Nanopore Technology (ONT) and/or Pacific Biosciences (PacBio) for long-read sequencing, along with Hi-C sequencing for chromosomal architecture, Illumina Paired-End (PE) for polishing (i.e. recommended for ONT-only assemblies), and RNA sequencing for transcriptomic profiling, to facilitate genome assembly and annotation.

### Sample and sampling information

On 08 July 2020, Aida Verdes and Patricia Alvarez-Campos sampled eight specimens of *Lineus lacteus* (sex unknown). The specimens were identified by Aida Verdes *in visu* in the Isla de Tabarca, Spain. The biological material collected in Spain, and used to generate digital sequences, was retrieved from wildlife taxa regulated by the Spanish Royal Decree 124/2017 ( https://www.boe.es/eli/rd/2017/24/124). The specimens were preserved alive by Patricia Alvarez-Campos until DNA extraction. Specimen were kept in small tanks with artificial sea water inside an incubator at 20 °C and fed frozen liver pieces weekly.

### Vouchering information

Physical reference materials for the sequenced specimen here have been deposited in the Museo Nacional de Ciencias Naturales (MNCN) https://www.mncn.csic.es/en under accession number MNCN 5.03/7.

Frozen reference tissue material from the posterior end of the body is also available from a proxy voucher at the Biobank of the Museo Nacional de Ciencias Naturales (MNCN) https://www.mncn.csic.es/en under voucher ID MNCN-ADN-151756 and from vouchers at the Biobank of the Leibniz Institute for the Analysis of Biodiversity Change https://leibniz-lib.de/en/ under voucher IDs ZFMK-TIS-102878—102938, ZFMK-DNA-FD19596985, ZFMK-RNA-FD19597035—19597036.

### Data availability

*Lineus lacteus* and the related genomic study were assigned to Tree of Life ID (ToLID) ‘tnRamLact8’ and all sample, sequence, and assembly information are available under the umbrella BioProject PRJEB77793. The sample information is available at the following BioSample accessions: SAMEA114541005, SAMEA114541015, and SAMEA117738716. The genome assembly is accessible from ENA under accession number GCA_965152395.1. The annotated genome will be made available through the Ensembl website ( https://projects.ensembl.org/erga-bge/). Sequencing data produced as part of this project are available from ENA at the following accessions: ERX13168324, ERX13168323, ERX14048567, and ERX12752257. Documentation related to the genome assembly and curation can be found in the ERGA Assembly Report (EAR) document available at https://github.com/ERGA-consortium/EARs/tree/main/Assembly_Reports/Lineus_lacteus/tnRamLact8. Further details and data about the project are hosted on the ERGA portal at https://portal.erga-biodiversity.eu/data_portal/947578.

### Genetic information

The estimated genome size, based on ancestral taxa, is 1.37 Gb. This is a diploid genome with a haploid number of 16 chromosomes (2n=32). All information for this species was retrieved from Genomes on a Tree ( Challis *et al.*, 2023).

### DNA/RNA processing

DNA was extracted from mid body using the Blood & Cell Culture DNA Midi Kit (Qiagen) following the manufacturer’s instructions. DNA quantification was performed using a Qubit dsDNA BR Assay Kit (Thermo Fisher Scientific), and DNA integrity was assessed using a Genomic DNA 165 Kb Kit (Agilent) on the Femto Pulse system (Agilent). The DNA was stored at 4 °C until used.

RNA was extracted using a RNeasy Mini Kit (Qiagen) according to the manufacturer’s instructions. RNA was extracted from two different specimen parts: head and mid body. RNA quantification was performed using the Qubit RNA BR kit and RNA integrity was assessed using a Bioanalyzer 2100 system RNA 6000 Pico Kit (Agilent). The RNA was stored at -80 °C until used.

### Library preparation and sequencing

For long-read whole genome sequencing, a library was prepared using the SQK-LSK114 Kit (Oxford Nanopore Technologies, ONT), which was then sequenced on a PromethION 24 A Series instrument (ONT). A short-read whole genome sequencing library was prepared using the KAPA Hyper Prep Kit (Roche). A Hi-C library was prepared from posterior body tissue using the Dovetail Omni-C Kit (Cantata Bio), followed by the KAPA Hyper Prep Kit for Illumina sequencing (Roche). The RNA library was prepared from an equimolarly pooled sample using the KAPA mRNA Hyper prep kit (Roche). The short-read libraries were sequenced on a NovaSeq 6000 instrument (Illumina). In total 339x Oxford Nanopore, 274x Illumina WGS shotgun, and 64.6x HiC data were sequenced to generate the assembly.

### Genome assembly methods

The genome was assembled using the CNAG CLAWS pipeline ( Gomez-Garrido, 2024). Briefly, reads were preprocessed for quality and length using Trim Galore v0.6.7 ( http://www.bioinformatics.babraham.ac.uk/projects/trim_galore/) and Filtlong v0.2.1 ( https://github.com/rrwick/Filtlong), and initial contigs were assembled using NextDenovo v2.5.0 ( Hu *et al.*, 2024), followed by polishing of the assembled contigs using HyPo v1.0.3 ( Kundu *et al.*, 2019), removal of retained haplotigs using purge-dups v1.2.6 ( Guan *et al.*, 2020) and scaffolding with YaHS v1.2a ( Zhou *et al.*, 2023). Finally, assembled scaffolds were curated via manual inspection using Pretext v0.2.5 with the Rapid Curation Toolkit ( https://gitlab.com/wtsi-grit/rapid-curation) to remove any false joins and incorporate any sequences not automatically scaffolded into their respective locations in the chromosomal pseudomolecules (or super-scaffolds). Summary analysis of the released assembly was performed using the ERGA-BGE Genome Report ASM Galaxy workflow ( 10.48546/workflowhub.workflow.1103.2).

## Results

### Genome assembly

**Figure 1.**
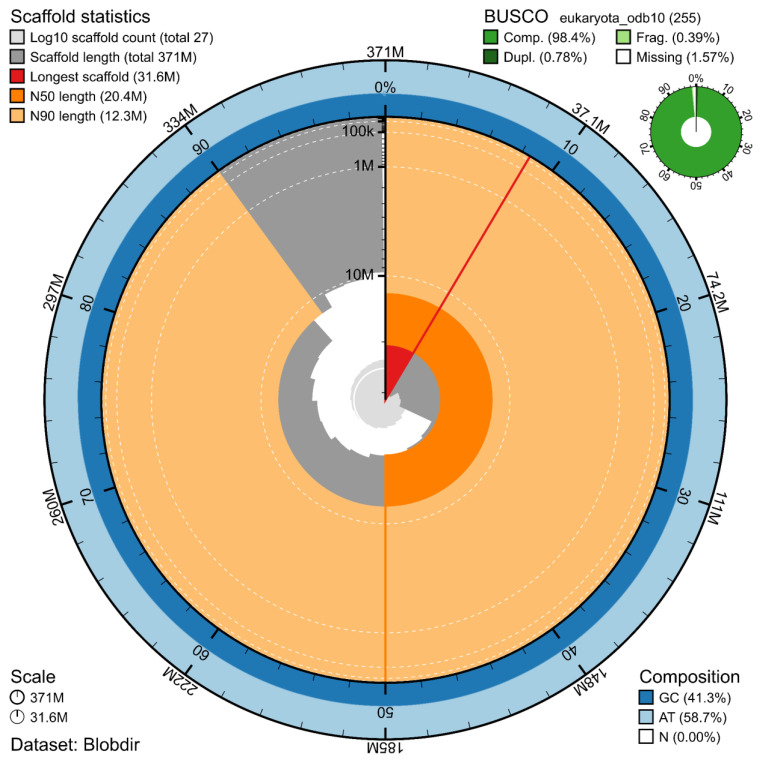
Snail plot summary of assembly statistics. The main plot is divided into 1,000 size-ordered bins around the circumference, with each bin representing 0.1% of the 370,829,418 bp assembly. The distribution of sequence lengths is shown in dark grey, with the plot radius scaled to the longest sequence present in the assembly (31.6 Mb, shown in red). Orange and pale-orange arcs show the scaffold N50 and N90 sequence lengths (20,380,758 and 12,283,121 bp), respectively. The pale grey spiral shows the cumulative sequence count on a log-scale, with white scale lines showing successive orders of magnitude. The blue and pale-blue area around the outside of the plot shows the distribution of GC, AT, and N percentages in the same bins as the inner plot. A summary of complete, fragmented, duplicated, and missing BUSCO genes found in the assembled genome from the Eukaryota database (odb10) is shown in the top right.

**Figure 2.**
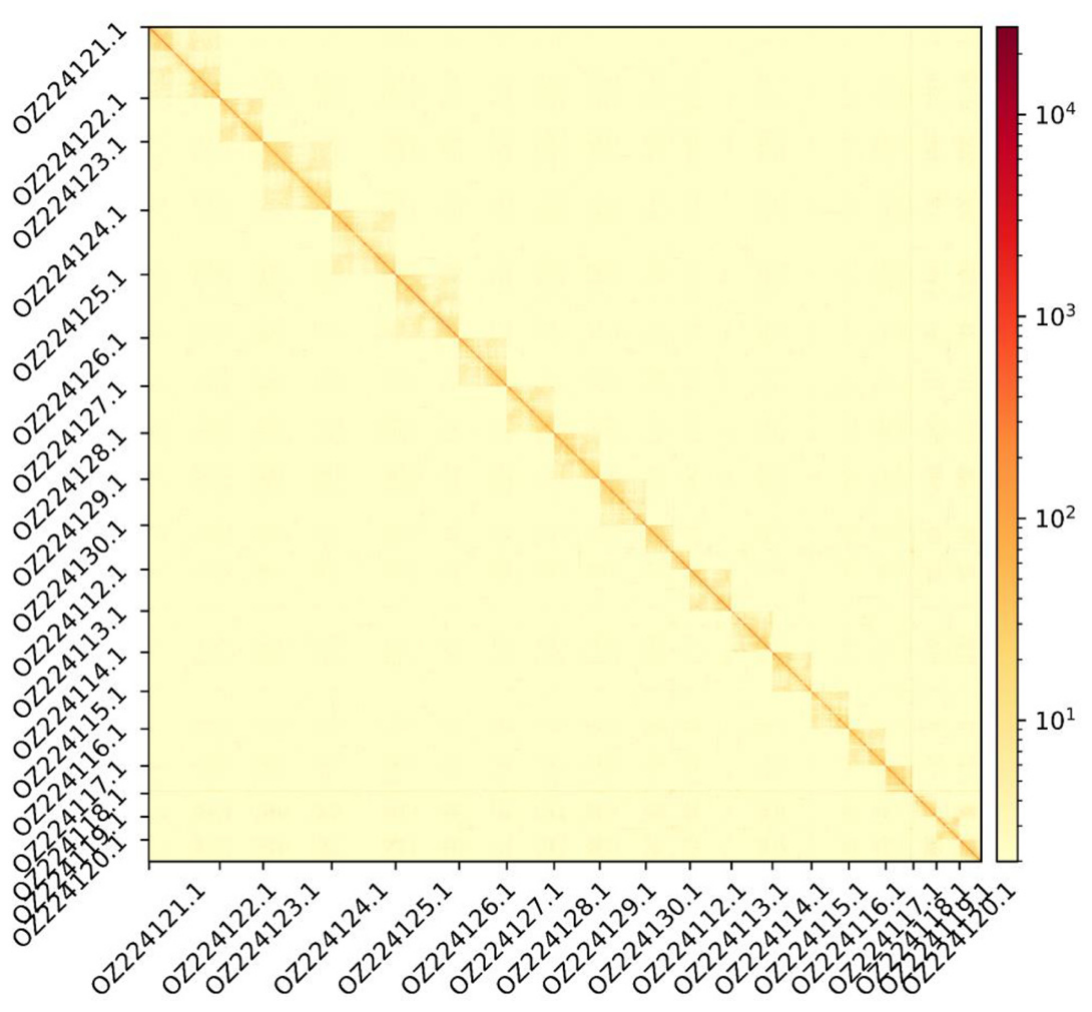
Hi-C contact map showing spatial interactions between regions of the genome. The diagonal corresponds to intra-chromosomal contacts, depicting chromosome boundaries. The frequency of contacts is shown on a logarithmic heatmap scale. Hi-C matrix bins were merged into a 100 kb bin size for plotting.

The genome assembly has a total length of 370,829,418 bp in 19 contiguous chromosomes ( Figure 1 & Figure 2), with a GC content of 41.3%. The assembly has a contig N50 of 8,945,886 bp and L50 of 14 and a scaffold N50 of 20,380,758 bp and L50 of 8. The assembly has a total of 44 gaps, totalling 8.8 kb in cumulative size. The single-copy gene content analysis using the Eukaryota database with BUSCO ( Manni *et al.*, 2021) resulted in 98.4% completeness (97.6% single and 0.8% duplicated). 76.4% of reads k-mers were present in the assembly and the assembly has a base accuracy Quality Value (QV) of 42.6 as calculated by Merqury ( Rhie *et al.*, 2020).
