# Supplementary material for: ERGA-BGE reference genome of the lineid heteronemertean Lineus lacteus (Pilidiophora, Nemertea)
Source: Open Res Eur. 2025 Nov 25;5:317. Originally published 2025 Oct 16. [Version 2] doi: 10.12688/openreseurope.21501.2 (PMC12816949; doi:10.12688/openreseurope.21501.2)
Supplement: Peer review history

# Reviewer report: Reviewer response for version 2

**DOI:** 10.21956/openreseurope.23793.r67370

**Published:** 2026-01-19

**Authors:** Natsumi Hookabe

**Affiliations:** 1 Japan Agency for Marine-Earth Science and Technology (JAMSTEC), Yokosuka, Japan

The manuscript entitled “ERGA-BGE reference genome of the lineid heteronemertean *Lineus lacteus* (Pilidiophora, Nemertea)” by Verdes et al. is clearly written and presents high-quality genomic data. The methods are sound, the results robust, and the resource valuable for future nemertean and comparative genomic studies. The manuscript is acceptable as submitted.

Are sufficient details of methods and materials provided to allow replication by others?

Yes

Is the rationale for creating the dataset(s) clearly described?

Yes

Are the datasets clearly presented in a useable and accessible format?

Yes

Are the protocols appropriate and is the work technically sound?

Yes

Reviewer Expertise:

Systematics; Phylogeny; Nemertean biology

I confirm that I have read this submission and believe that I have an appropriate level of expertise to confirm that it is of an acceptable scientific standard.

# Reviewer report: Reviewer response for version 1

**DOI:** 10.21956/openreseurope.23257.r62661

**Published:** 2025-11-10

**Authors:** Thomas D Lewin

**Affiliations:** 1 Biodiversity Research Center Academia Sinica, Taipei City, Taipei City, Taiwan

Verdes et al. present a chromosome-level genome for the nemertean *Lineus lacteus*. Nemerteans are a phylum of vermiform animals within the Lophotrochozoa that is significantly underrepresented among genomic databases compared to related phyla like molluscs and annelids. This new nemertean genome will therefore be of great value to the study of lophotrochozoan genomics and evolution. In particular, the authors highlight the potential for insights into venom production and regeneration given the prevalence of these traits within nemerteans. The genome itself is of high quality and the article is the standard format for a “genome note” style publication. It is well-written and contains all of the necessary information and explanation. The sequencing data and assembled genome are made available for public use. I have no concerns about the quality of the work or the genome and strongly support its indexation.

 I have only a couple of very minor comments that the authors may wish to consider.

  

1. I would expect the genome to have undergone some kind of decontamination procedure as part of the assembly process, such as with BlobToolKit (Challis et al. 2020 G3), but as it stands I can’t see any description of this. Was any decontamination step completed?
2. Currently the manuscript only reports that “the specimens were preserved alive … until DNA extraction”. It might be good to provide a little bit more information here. How long were they kept? What were the culture conditions? Etc etc. Anything that could be useful for someone else looking to do culture these animals.
3. The citation for purge_dups appears to be missing.

Are sufficient details of methods and materials provided to allow replication by others?

Yes

Is the rationale for creating the dataset(s) clearly described?

Yes

Are the datasets clearly presented in a useable and accessible format?

Yes

Are the protocols appropriate and is the work technically sound?

Yes

Reviewer Expertise:

Evolutionary genomics

I confirm that I have read this submission and believe that I have an appropriate level of expertise to confirm that it is of an acceptable scientific standard.

# Response

**Published:** 2025-11-20

**Authors:** Chiara Bortoluzzi

**Affiliations:** Swiss Institute of Bioinformatics, Lausanne, Vaud, Switzerland

In this new version of the Genome Report, we have addressed the reviewer's comments as below: 

1. The decontamination step was completed. We did not report these analyses in the main text, because these are already reported in the EAR, which we refer to in the main text using the following link: https://github.com/ERGA-consortium/EARs/tree/main/Assembly_Reports/Lineus_lacteus/tnRamLact8. 
2. We provide additional information regarding the maintenance of the specimens in the lab. 
3. We added the missing citation for the purge_dups software.
